# Can a personalised music listening intervention decrease agitation in hospitalised patients with dementia? A feasibility trial

**DOI:** 10.3389/fpsyt.2023.1186043

**Published:** 2023-08-08

**Authors:** Shanna Lee, Lily Chan, John Maddison

**Affiliations:** ^1^Adelaide Medical School, The University of Adelaide, Adelaide, SA, Australia; ^2^Riverland General Hospital, Riverland Mallee Coorong Local Health Network, Berri, SA, Australia; ^3^School of Public Health, The University of Adelaide, Adelaide, SA, Australia; ^4^Geriatric Medicine, Northern Adelaide Local Health Network, Adelaide, SA, Australia

**Keywords:** music, dementia, personalised playlist, non-pharmacological intervention, agitation

## Abstract

**Introduction:**

Agitation is a common manifestation of the behavioural and psychological symptoms of dementia (BPSD). Pharmacotherapy is not the first-line management because of its potential harms, particularly in the elderly. Music as a non-pharmacological intervention for agitation has been explored in residential aged-care facilities, but few studies have been situated in hospitals. This pilot aims to evaluate the feasibility of a personalised music listening intervention for reducing agitation in hospitalised patients with dementia in a metropolitan Geriatric Evaluation and Management (GEM) unit.

**Methods:**

Two-arm randomised control feasibility trial. Eligible patients were assigned to the music intervention or control group, with the intervention group receiving music daily between 15:00–16:00, and agitation levels measured in both groups hourly based on the Pittsburgh Agitation Score (PAS) over 5 days of hospitalisation. Post-trial semi-structured interviews assessed feasibility of the intervention.

**Results:**

Twenty-one patients were recruited over 8 months. Interviews with staff involved indicated that the music intervention was manageable to deliver, assisted engagement with patients which increased efficiency of some clinical tasks, and challenged staff mindset around using psychotropic medication to address agitation. PAS results were inconclusive, because of underpowered numbers in this pilot study.

**Conclusion:**

It is feasible for nursing staff to deliver a personalised music listening intervention to patients with dementia in a geriatric unit of a tertiary hospital, without compromising on usual clinical care.

## 1. Introduction

Agitation is a term used to describe a cluster of verbal and motor behaviours that are inappropriate in nature, frequency or social standards (1), and is a common manifestation of the behavioural and psychological symptoms of dementia (BPSD). Reframed outside of a disease paradigm, agitation can be understood as “reactions to unmet psychosocial needs, and therefore attempts to communicate these needs and as ways to cope” (1). Current management of BPSD includes non-pharmacological and pharmacological methods, the latter typically including antidepressants, anticonvulsants, benzodiazepines and antipsychotics. Yet, psychotropics have mixed and limited evidence, and are not without risk, potentially resulting in unfavourable side effects and harms with use (2, 3). Thus, the use of non-pharmacological strategies may reduce the subsequent risks of pharmacological interventions, and indeed is the first-line approach for the management of BPSD (4–6).

There is increasing evidence for the use of music as a non-pharmacological intervention to address BPSD. There has been a breadth of benefits reported in the literature worldwide, including a significant reduction in anxiety and agitation (7, 8), short-term improvement in behaviour and mood (9, 10), and enhanced participation in activities by reducing apathy (4) and facilitating social interaction (3). Music has the capacity to enhance functional abilities (11) by providing an “interpretable stimulus” (12) which increases attention and regulation to surrounding environments (3, 11). The use of personally significant music may increase the threshold at which people with dementia can tolerate unfamiliar environments, because of perceived meaning and stimulation of memory (3, 4). Despite evidence suggesting that music has positive short-term effects, reviews have concluded its efficacy in addressing behavioural difficulties in dementia as “inconclusive” (13) mainly due to low to moderate-quality evidence presented (14). Additionally, most studies of this nature have taken place in the context of residential aged care facilities (RACF), with limited studies in hospitals (3).

Personalised, or individualised, music is defined as music that is preferred prior to the onset of cognitive impairment, which is generally derived from one’s young adulthood years (3, 11). Despite declines in cognition, the literature indicates that the neurological areas that respond to music are relatively preserved and deteriorate later in the disease process (15, 16). Music can thus stimulate and elicit memories with associated positive feelings (17). There are a few caveats, notably that the positive effect of music is not universal. This may be due to music’s relative insignificance for the individual prior to cognitive impairment (i.e., it is not a universally significant medium); the subjective experience of music as listened to and processed from one day to the next (i.e., a person’s emotions/experience of the day have a bearing on the emotional impact of music listening) (18) and the time of day that music is played (3, 19). Thus, the purpose of this pilot study is to investigate the feasibility of a personalised music listening intervention (PMLI) in decreasing agitation in a hospital setting given the gap in the literature regarding the use of music in this context. The merits and feasibility of the intervention will primarily be examined on parameters such as ease of protocol delivery, compliance, staff receptivity and adequate resources, in addition to analysing the PMLI effect on participants (20, 21).

## 2. Methods

### 2.1. Trial design

This was a single-centre, 2-arm (1: 1 ratio), parallel-group, randomised control feasibility trial. This study was approved by CALHN Human Research Ethics Committee (HREC/19/CALHN/200) and conducted in full conformance with principles of the “Declaration of Helsinki,” Good Clinical Practice and within the laws and regulations of Australia. Given the aim, a descriptive phenomenology approach was used to assess PMLI feasibility from the intervention facilitators’ perspective (12, 19). The primary investigator has undertaken two research projects prior to this hospital pilot, an observational case study in 2015 and community based participatory research in 2018, based in a RACF (22, 23). These studies were framed around the key concept utilised in this pilot, that the use of personalised music playlists tend to confer a benefit to listeners with dementia, in keeping with the wider literature of studies in RACF settings (3, 4, 7–10). The methods from previous projects were adapted for this pilot, particularly questionnaires (19).

### 2.2. Context and participants

The study was undertaken in a 24-bed Geriatric Evaluation and Management (GEM) unit in a metropolitan hospital in Adelaide, Australia. All admitted patients aged 65 years and over, or 50 years and over from an Aboriginal or Torres Strait Islander background (24, 25), and admitted to the GEM unit with a diagnosis of dementia were screened for enrolment based on the below criteria. This was undertaken collaboratively by the investigator and Nurse Unit Manager (NUM).

Detailed inclusion and exclusion criteria were established to simulate full trial conditions, however the working definition for exclusion was the inability to engage in a conversation for the preliminary interview to create the participant’s personalised playlist. This was determined by the NUM. Inclusion criteria: admission with an anticipated length of stay greater than five or more days and ability to communicate verbally. Exclusion criteria: patients readmitted to GEM unit following completion of study protocol on index admission, patients with severe dementia and/or delirium with difficulty in verbal communication, a significant language barrier, and significant psychiatric diagnosis requiring pharmacological treatment. Note, the latter did not include diagnoses like depression if it did not significantly impact function (i.e., the ability to engage appropriately with the semi-structured interview, and verbalise music preferences).

All participants were provided with an information sheet. Consent was given by participants. If this was not possible, consent was given by next of kin in accordance with approved research ethics approval.

Participants were purposefully allocated to either the intervention or control group in an alternating sequence by the NUM (i.e., first patient enrolled – intervention group, second patient enrolled – control group etc.). During the period of data collection, there were changes to the NUM for reasons including annual leave and secondment, which accounts for the slight discrepancy in the numbers of participants recruited per trial arm. Blinding was not possible because of the nature of the music intervention, as nursing staff were facilitators of the intervention whilst being directly involved in patient care regardless of whether the participant was in the intervention or control group.

### 2.3. Intervention

The intervention was a music playlist of personally curated songs using an online music listening platform. The nature of the PMLI reflects Gardner’s protocols (19) which have been used in other studies (3, 18) in addition to the investigator’s prior residential aged care facility case studies in 2015 and 2018 (22, 23).

It is important to note early in this paper that “music” is a broad term and, in this field, there are guides to appropriately specify its use (26). In this study, the use of music was: personalised, based on Gerdner’s mid-range theory of individualised music interventions for agitation (19); receptive, rather than actively playing music with instruments or singing; with individuals listening to music rather than in group settings (21), and playing pre-recorded music from an electronic device, rather than being facilitated by a formal music therapist. Those enrolled in the intervention group underwent a semi-structured interview in a private room of the ward with a member of the research team (the primary investigator, hospital volunteer or allied health assistant) to establish the participant’s music preferences. A standardised questionnaire informed by Gardner’s was used to obtain preferred and significant songs for participants (12). Once identified, these pre-recorded songs were added to their playlist. The second part of this interview consisted of playing 30 s snippets of songs from a selection of nine genre-specified playlists directed by preferences ascertained by questionnaire responses. These playlists were created by the investigator prior to patient recruitment and consisted of the following categories: Classical, Musicals, Christian, 20s 30s 40s (Jazz), 50s (Rock n’ Roll/Blues), 60s (Motown/Soul) 70s (Groove/Disco), 80s 90s (Classic Hits) and Contemporary. Participants would verbally indicate to the interviewer their preference for the song, which would then be added to their personalised playlist, with the aim of creating a playlist of approximately 50 min duration.

Participants listened to their playlist daily between 15:00–16:00 for five consecutive days. The duration of the intervention was chosen due to the average length of stay in the unit, for sufficient data to be captured before discharge. The time was chosen because of the “sundowning effect,” when agitated and anxious behaviours are generally exhibited, as theorised by the Progressive Lowered Stress Threshold model (27). In addition to the scheduled daily sessions, music could be play on an as required basis, initiated either by participants or nursing staff, in order to target music delivery to individual need (3, 11, 28).

The five iPads used for the interviews were sanitised for infection prevention before and after each use. The intervention was delivered by nurses or ward volunteers in individual rooms, or if the patient was in a bay, curtains were drawn to minimise ambient sound. Treatment fidelity was ensured by the primary investigator providing a training video indicating how to create and add songs to playlists, and printed protocol instructions which the NUM relayed to nursing staff.

The control group received routine medical care expected for GEM patients.

### 2.4. Sample size

A sample size of 60 patients was determined to be suitable for detecting significant impacts on outcome measures (i.e., PAS, CGI) in the context of a novel intervention undertaken in a metropolitan hospital ward. Despite the study being terminated before the intended sample size was reached, due to difficulties experienced during the COVID-19 pandemic, the actual sample size (*n* = 19) was deemed acceptable since the aim of this study was feasibility of the music intervention within the unit.

### 2.5. Data collection methods

The primary outcome, feasibility, was assessed by 45 min semi-structured interviews post-trial with five key members of the intervention delivery and oversight (GEM Nurse Unit Manager, nursing and allied health staff, hospital volunteers). Because staff facilitated the intervention, their assessment was crucial to determine feasibility. Questions covered staff satisfaction with the intervention and their assessment of the intervention as feasible and sustainable in the GEM unit based on staff, patient, environmental and study design factors. Interviews were conducted over *Zoom* due to COVID-19 limitations and initial handwritten notes taken by the primary investigator were typed and thematically analysed.

Nurses were also given the option to write observational notes at hourly intervals, to explain what behaviours were observed and how, if relevant, the intervention addressed agitated behaviours. This provided qualitative data that extended beyond time-point measures to assist in PAS interpretation at specific points in time.

Quantitative data was also collected in this study for several purposes. First, given this was a feasibility trial, full test conditions were imitated to assess the pragmatics of the data collection process. Second, the data was used to triangulate with interviews, to analyse for harms and opportunity costs. Quantitative measures collected include the Pittsburgh Agitation Scale (PAS) and the Clinical Global Impression (CGI).

The PAS is a brief measure of agitation that measures the severity of agitation in four general categories: aberrant vocalisation, motor agitation, aggressiveness and resisting care, on a scale from 0 (not present) to 4 (highest level) (29). The score from each category reflects the most severe behaviour within each behaviour group. Scores from each category are added to form a total PAS score, hence the range of PAS is 0–16 points. The scale was administered by direct observation of the participant by nursing staff, scored hourly from day of admission (as a baseline measure of agitation) and a further 5 days. PAS was the chosen instrument because it is a validated tool for assessing agitation and coincidentally, was already being utilised at hourly frequencies for a concurrent pilot project occurring in the GEM unit, thus making it pragmatic for nursing staff to administer. Although PAS was collected in hourly intervals, the investigator translated PAS data in 2 hourly blocks for convenience of data transcribing. Given that analysis of intervention efficacy was not the primary aim of this feasibility trial, having fewer data points for statistical analysis was acceptable.

This pilot study used two of the three questions in the CGI to assess intervention effect (30). The first question assessed the severity of a patient’s clinical condition before the intervention, with a standardised response selected from a Likert scale from 1 (normal, not at all ill) to 7 (among the most extremely ill patients). This was used to capture baseline characteristics (Table 1). The second question assessed global improvement, with a similar standardised response selected from a Likert scale of 1 (very much improved) to 7 (very much worse). The CGI was completed by the same clinician for patients pre- and post-trial whether in the intervention or control group and was thus intended to demonstrate the positive role of music (if present) by a more favourable post study CGI score.

**Table 1:** Baseline characteristics of study participants.

	Music intervention group (*n* = 12)	Control group (*n* = 9)
Age in years (mean, sd)	82.4 (6.3)	84.7 (6.7)
(median, IQR)	84.0 (78.0–87.0)	88.0 (81.0–89.0)
Male (*n*, %)	6 (50)	3 (33)
Charlson Comorbidity Index^a^ (mean, sd)	2.0 (1.7)	1.9 (1.7)
(median, IQR)	1.0 (1.0–2.0)	1.0 (1.0–2.0)
Cumulative Illness Rating Scale^a^ (mean, sd)	10.9 (4.1)	7.7 (2.1)
(median, IQR)	11.5 (8.0–13.0)	8.0 (7.0–9.0)
Clinical Frailty Index^a^ (mean, sd)	5.7 (1.8)	5.6 (0.9)
(median, IQR)	6.0 (6.0–7.0)	6.0 (5.0–6.0)
Mini-Mental State Exam Score^b^ (mean, sd)	19.5 (3.6)	20.1 (5.0)
(median, IQR)	20.0 (17.0–22.0)	21.0 (18.0–23.0)
Clinical Global Impression^c^ (Day 1) (mean, sd)	3.9 (0.5)	3.9 (1.3)
(median, IQR)	4.0 (4.0–4.0)	4.0 (4.0–5.0)

a missing *n* = 2 in music group.

b missing *n* = 1 in music group.

c Clinical Global Impression (Day 1) measured severity of mental illness.

Additional outcomes pertaining to feasibility and intervention impact included: patient/carer satisfaction with the intervention and reduction in the use of psychotropic medication in intervention group. Although intended to be assessed by means of qualitative survey, interviews and review of medication charts, these outcomes were not evaluated. This was largely due to the limited resources of the investigator undertaking the pilot (i.e., no capacity to hire research staff dedicated to collecting additional data beyond the primary PAS measure). Additionally, a site-specific limitation that contributed to difficulties in assessing psychotropic medication use was the lack of electronic medical records, which would have assisted evaluation of patient drug use with logged data in time/date form.

### 2.6. Data analysis

Interview responses were coded by one author, with initial findings communicated to one other co-author for feedback. Given the goal of this study was to assess feasibility, data was inductive and descriptively coded to explore the breadth of respondents’ views considering the therapeutic use of music in a hospital context. Codes were then collated based on similarities in inferences to create the following themes: “Music connects people with one another and their tasks” and “Music challenges the usual running of a geriatric ward”. Nursing PAS notes were not coded and thus not integrated into the thematic analysis. Rather, they were referred to when, upon analysis of quantitative PAS data, spikes in PAS were recorded. Then, nursing notes were referenced to corroborate and shed further light on the PAS data.

Just as quantitative data was collected in a manner to emulate a full trial, use of statistical models for analysis were implemented to test their feasibility and fit for future research. Data are presented as mean (standard deviation, SD) and as median (interquartile range, IQR) for continuous variables; and as count and percentages (%) for categorical variables. A multilevel random-intercept negative binomial regression model was used to examine if the 2-hourly PAS scores over the 5 days were different between the intervention and control groups. A negative binomial regression model was chosen to allow for over-dispersion of data. A multilevel model approach was used in order to account for the correlation between nested data (2-hourly PAS nested within individuals). Several possible models including treatment group, covariates of day of study and age (both as continuous variables), and/or gender were examined, and their Akaike Information Criterion (AIC) compared. The final selected model was the one with the lowest AIC. As the lasting effect of music listening was unclear but likely to be short-lived, a second exploratory analysis was performed examining just the PAS scores obtained each day for the period between 12:00 to 14:00 (pre-music listening, Time 1) and between 16:00 to 18:00 (post-music listening, Time 2) (9, 10). Again, several possible models including treatment group and time, group by time, and covariates of day, age, and/or gender were examined and the model with the lowest AIC was selected. Wilcoxon rank-sum test was used to compare Clinical Global Impression (CGI) rating on the last day of the trial (Day 5) between treatment groups. The two-tailed significance level was set at α = 0.05. All statistical analyses were performed using Stata/IC 15.1 (StataCorp LLC, College Station, Texas, United States).

## 3. Results

**Figure 1:**
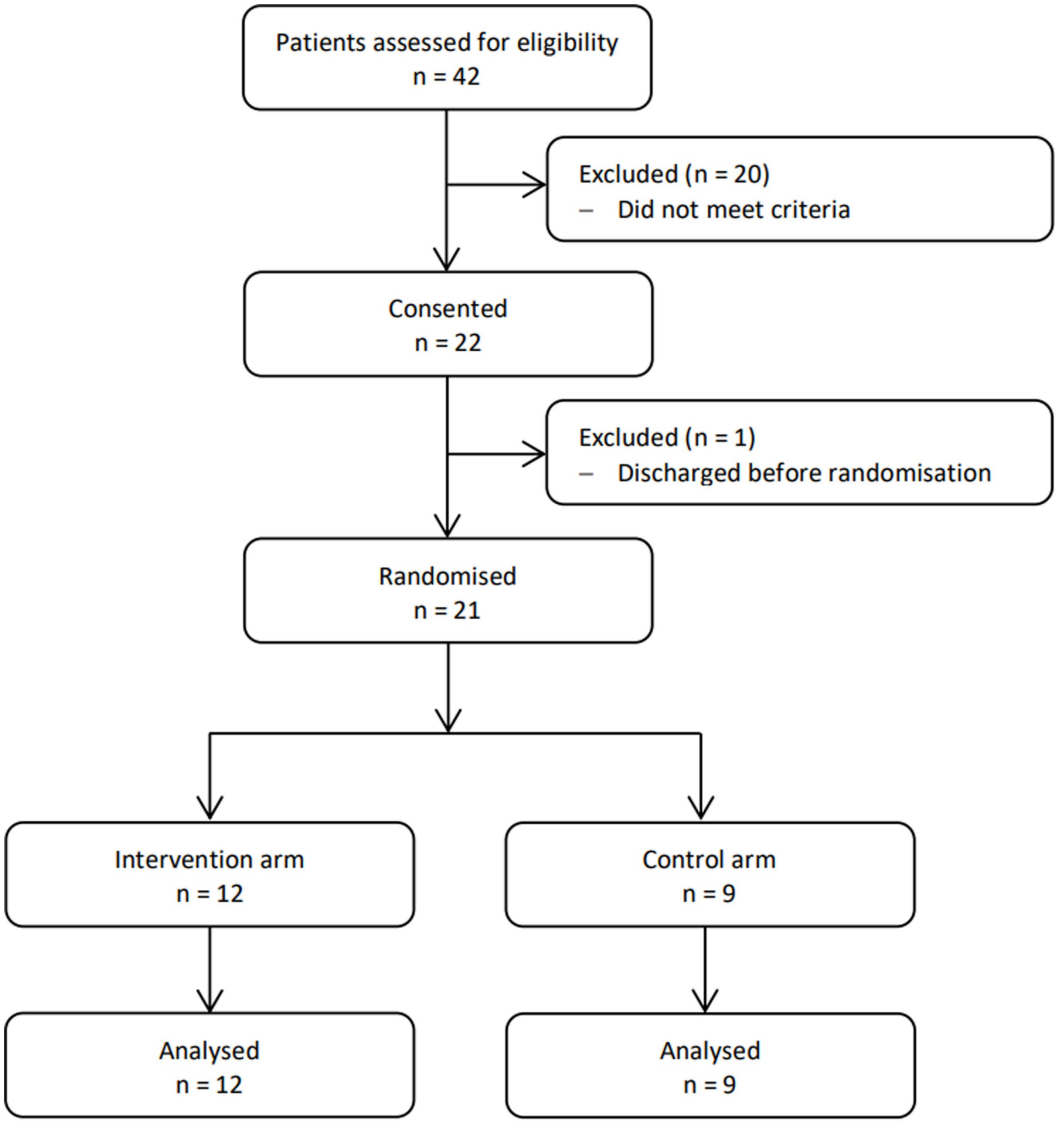
CONSORT diagram showing the flow of participants through each stage of the trial.

Recruitment occurred from July 2019 – March 2020. It was anticipated that 6 months was an adequate trial duration to enrol the figure of 60 patients. However, in the 8 months duration of this pilot, 42 patients were assessed for eligibility and 20 were excluded (Figure 1). 1 participant was withdrawn from the study because they were discharged from GEM before a minimum of 5 days of data collection. Additionally, the decision was made to stop enrolment and data collection because of the impact of COVID-19, which halted ongoing research trials in the hospital. Table 1 outlines the demographic data of study participants.

### 3.1. Music connects people with one another and their tasks

A common occurrence in interviews was that use of the PMLI generated a connection between staff and patients:

“[staff] loved the program because they got to engage with patients” (N2, Assistant Nurse Unit Manager)

“[there was] opportunity to sing and dance with patients” (N1, Nurse Unit Manager)

“sometimes [when] everyone was singing like it was a jam session – nurses smile and everyone is in a positive mood” (V1, hospital volunteer)

The nature of this connection was expressive, with comments indicating how music evoked observable positive responses in patients and staff:

“[music had] a very good impact on patients – their reactions were smiling, happy, drumming hands on table…even for patients who may be initially grumpy, as soon as they heard the music their personality improves” (V1)

“I feel so happy and fulfilled because it makes their [patients’] day” (V1)

“…seeing the enjoyment on patient’s faces” (N1)

“[music] decreases stress [for staff]” (N3, Assistant Nurse Unit Manager)

Beyond the initial, reactive, emotional impact on individuals, there were examples given of “practical” impacts of music on patients, including observable decreases in agitative behaviours. Several comments suggest that this granted staff the ability to better attend to their clinical duties with greater focus.

“[music was a] short term alleviation to boredom” (N1)

“music generally made patients more relaxed, settled into routine and more involved in other ward activities … [music] helped to meet a patient’s care needs in a positive way,” (N3)

“[every time] Amazing Grace was played they calmed down – they usually had wandering behaviours – it decreased agitation and increased their recollection … music assisted patient engagement with nurses … [they were] more efficient with their job” (V2, allied health assistant)

“music helped in delirium to de-escalate PAS and 4AT, also in patients with BPSD” (N3)

In view of feasibility, these latter interview responses are indicative of GEM staffs’ positive reception to a PMLI within their unit. Acceptance of the intervention may be due to music’s ability to facilitate the delivery of clinical care and duties. Factors that contributed to the uptake of the PMLI, and thus its acceptability and feasibility as an intervention from the staff perspective, are summarised by this comment (factors bolded):

“[the PMLI] was manageable [to facilitate] especially when volunteers were **trained**. Additional **resources provided** such as the ‘how to make a playlist’ video helped. **Protocols** provided structure. Playlists were a tangible tool – set up and ready to go” (N1)

Finally, all respondents agreed that the intervention was suited for the GEM unit, and suggested additional locations where they identified the intervention having potential benefits (older persons’ mental health unit, other medical wards, and nursing homes). This summarises the interviewees’ position that the PMLI was ultimately feasible in its current form.

### 3.2. Music challenges the usual running of a geriatric ward

On the other hand, interviewees also reported several challenges in the intervention’s utilisation within the GEM unit. These were across several categories, as Table 2 illustrates.

**Table 2:** Challenges with the personalised music listening intervention.

Categories	Interviewee responses
Patient	Getting consent (N1)
	Inconsistent “results” with the use of music. Some days it “worked” and others it did not (V2)
	Most patients responded well, but some did not – 1%. If patients were grumpy before the music, this was a challenge to engage them (V1)
Staff	Certain individuals feel like it was a chore (N2)
	Set up process – need to be tech savvy (N1)
	Understanding the intricacies of inclusion and exclusion criteria (N1)
	Communicating with staff which patients to approach for consent was tricky because they are time-poor (V1)
	Busy (N3)
	Inconsistency of staff (N3)
Ward environment	GEM unit itself – not designed for people with dementia (N1)
	Time – it’s a busy unit. Not enough time spent with patients (N1)
	When there are lots of patients around, the patient of focus can be distracted by others talking (V1)
	Ward traffic – distractions (N2)
	Not all patients wanted to listen to music…because of distractions (V2)
Study design	More paperwork to complete…reminding staff (N2)
	iPads were tricky to use if patients had infectious precautions (N3)
	Inclusion/exclusion criteria too specific, it could be broader to include all “cognitive impairment” rather than just dementia (N1)

There is a crossover in analysing the challenges across the categories of ward environment and staff, particularly from a feasibility perspective. This is because staff interact with one another and patients inside the constraints of the physical ward space, whilst working within the expectations of their existing roles and responsibilities. Yet, because staff deliver the intervention, they are key subjects to analyse for feasibility in answering the research question.

Comments revealed innate “pre-requisites” staff required to adequately facilitate the intervention including tech-savviness and an understanding of the study protocol including recruitment criteria. Additionally, based on the comment that the intervention was at times viewed as a chore, the implication is that the opposite is likely true, where a positive attitude towards the PMLI is key for staff acceptance. On the other hand, related to the ward structure were staff comments about busyness and being time-poor, in addition to the changeability of staff in a 24 h shift work environment.

In conjunction with reported ward traffic, small spaces and distractions, these, cumulatively framed with a feasibility lens, illustrate the contextual realities of this GEM unit, which this trial explored. Distractions were likely auditory in nature, extrapolating from interviewee comments. This could be a target for optimising feasibility in any future studies, by sourcing a quieter space to deliver the intervention in the ward or alternative hospital location, or trialling use of PMLI with headphones.

**Figure 2:**
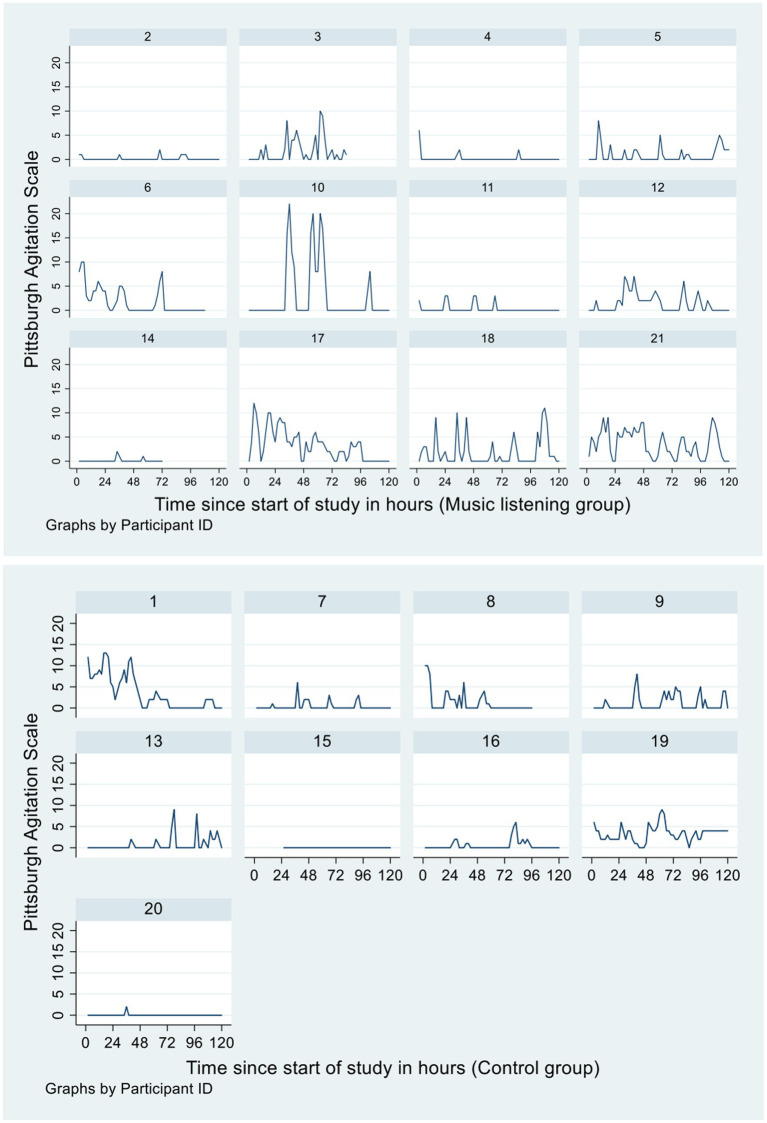
Observed 2-hourly Pittsburgh Agitation Scale scores over the 5-day study period.

The “inconsistency” of music’s effect conveys the nature of subjective experience, with qualitative nursing notes further demonstrating this phenomena (18). In this vignette, family members of Participant 5 (Figure 2) brought music from home as a known intervention to assist in their agitative behaviours. Days 1–3 comment: “music on, patient settled and continues to listen to own music,” “was listening to music and reading Bible,” “patient listening to music. Singing along with spouse, music playing in background for dinner. Singing along. Settled.” However, Day 4 states: “patient refused music at 15:00–16:00.” This endorses the quote that “some days it [music] “worked,” other days it did not” (V2).

Alternatively, the following vignette of Participant 6 (Figure 2) who had delirium secondary to a urinary tract infection illustrates a consistent reception to music across the 5 days of study participation in the intervention group: “listening to music helps in disbanding behaviour. Is calmed/settled” (Day 2), “participated in listening to music, displayed calm and settled behaviour” (Day 4), “can be confused at times, settled with reassurance. Continued to listen to music throughout the day, was settled” (Day 5). Although a positive illustration for the use of music, the case of Participant 6 demonstrates the challenge in establishing completely “quarantined” test conditions in relation to studying music under a purely scientific lens, particularly in a hospital context. The effect of music observed in an individual may be music itself acting directly, but also music interacting with many confounding factors.

Music challenged in another sense also. Two assistant NUMs reported decreases in PRN medication use for some patients, even in patients with extreme BPSD:

“[the study] opened staff’s eyes to use music as therapy. It triggered them to think of other ways to manage difficult behaviours; diversifying treatment before using PRNs [antipsychotics]” (N2)

However, they also recognised that patient behaviours did fluctuate, and was dependent on other factors, such as external triggers, previous mood, and activities of the day.

When asked about facets of the intervention that could be changed to improve any future implementation, “broadening the study design to include ‘cognitive impairment’ not just dementia” (N1) was suggested. This comment not only indicates possibility for increased patient involvement and uptake, but a willingness from a staff’s perspective that this would be feasible and beneficial. Additional comments supported the overall view of embracing the PMLI:

“shows that music is a universal thing” (V1)

“[music as] one of the options in the therapeutic toolkit” (V2)

“Music is a universal language – reminiscing and recollection” (V2)

Collectively, these interview responses are interesting because they suggest that, despite practical challenges, which affected the ease of delivering a PMLI in a hospital ward environment, there is a willingness from staff to embrace the intervention.

### 3.3. Quantitative data

#### 3.3.1. Outcome 1: Pittsburgh Agitation Scale scores

##### 3.3.1.1. Outcome 1.1: 2-hourly Pittsburgh Agitation Scale scores during the trial period

Figure 2 displayed the observed 2-hourly PAS scores of the 21 patients over the 5-day study period. A multilevel random-intercept negative binomial regression model was used to examine if the 2-hourly PAS scores over the 5 days were different between the intervention and control groups. The final selected model with the lowest AIC included randomisation group (intervention or control) as predictor and day of trial and gender as covariates. There was no significant main effect of randomisation (IRR 1.90, 95% CI: 0.57, 6.34, *p* = 0.299, control group as reference). The 95% CI for the predicted mean of the control group was 0 to 2.74 and for the intervention group was 0 to 5.17. The main effect of day was significant (IRR 0.83, 95% CI: 0.76, 0.91, *p* < 0.001). There was no significant main effect of gender (IRR 0.38, 95% CI: 0.11, 1.27, *p* = 0.116, female as reference). Interaction between randomisation and day was found to be not significant and therefore not included in the model.

##### 3.3.1.2. Outcome 1.2: daily pre- and post-music listening Pittsburgh Agitation Scale scores

The PMLI was delivered daily between 15:00–16:00 during the 5-day study period. A multilevel random-intercept negative binomial model was used to examine if the daily 2-hourly PAS scores recorded for the period between 16:00–18:00 (Time 2) were different to those recorded for the period between 12:00–14:00 (Time 1), and whether this difference, if any, differed between the two randomisation groups. The final selected model with the lowest AIC included randomisation group, time (Time 1 or Time 2) as predictors; and day, age and gender as covariates. The group by time interaction term was not significant and therefore removed from the model. There was no significant main effect of time (IRR 0.79, 95% CI: 0.47, 1.31, *p* = 0.355, Time 1 as reference); nor significant main effect of randomisation (IRR 2.78, 95% CI: 0.82, 9.43, *p* = 0.101, control group as reference) after adjusting for day, gender and age.

#### 3.3.2. Outcome 2: Clinical Global Impression rating

The mean and median CGI rating on Day 5 for the intervention group was 2.8 (SD 0.9) and 3.0 (IQR 2.0–3.0) respectively. The mean and median CGI rating on Day 5 for the control group was 3.3 (SD 0.9) and 4.0 (IQR 3.0–4.0) for the control group, respectively. There was no statistically significantly difference in CGI rating as assessed by Wilcoxon rank-sum test (*z* = 1.652, *p* = 0.099) although 11 out of 12 patients (92%) in the intervention group were rated as either “minimally improved” or “much improved” compared to 4 out of 9 patients (44%) in the control group.

### 3.4. Harms

To the investigator’s knowledge, there were no unintended effects or harms to participants in either arm of the trial.

## 4. Discussion

### 4.1. Interpretation

The overarching impression from interviews was that the intervention was feasible and satisfying to deliver. Reasons for this included: the provision of education, training and resources to nurses prior to trial commencement to enable familiarity with protocols, and staff belief in the value and benefits of music in this disease context. A potential source of bias in interpreting feasibility from a staff point of view is the gaining popularity and acceptance of the therapeutic use of music with older persons with dementia in the wider population, as indicated by comments positing music’s “universal” influence. This may have influenced the staffs’ perspectives towards an acceptance of the music intervention, despite practical challenges (21). One such challenge was the “inconsistent” effects of music, which could be due to prior mood and events of the day. Interestingly, Australian guidelines published recently for use of music for people with dementia in RACF suggest creating multiple playlists to respond to varied moods and behaviours (31). The recommendation may address this issue, but is beyond the scope of this feasibility trial.

Qualitative data demonstrated several further positive outcomes, including challenging the way psychotropic medications are currently used to address BPSD in GEM patients and increasing staff engagement with patients in ways that assist patient care and increase positive mood. The Hawthorne effect may explain this, as nursing staff were aware of their active role in facilitating the intervention, in addition to patients not being blinded to either group. This is not necessarily an inferior finding, for if staff perspectives and approaches to use of medications have changed because of self-awareness, this may benefit patients admitted to the GEM unit beyond the feasibility pilot itself as this attitude becomes integrated into personal practice.

Quantitative data collected in this trial indicate that PAS was higher in the intervention group than the control group. Despite data not significantly suggesting that a PMLI would be an effective means of reducing agitation in hospitalised patients with dementia, this should be interpreted as an inconclusive result rather than having no evidence for an effect, due to underpowered numbers as a pilot. Moreover, the intention of collecting quantitative measures was not to prove intervention efficacy, but to imitate full trial conditions in the real-world context, and to assess for potential harms. Triangulated with interview findings, PAS and CGI suggest that the intervention did no harm in this pilot.

### 4.2. Limitations

Several limitations have been identified in the pilot which have likely impacted upon the precision of results.

Targeted, specific inclusion criteria allowed the pilot to test the feasibility of and adherence to music protocols. However, this is a limitation because the excluded population were likely to experience and exhibit significant agitation; in other words, they had an arguably greater need for novel agitation management, and hence, there was a potential benefit missed.

The PAS is an objective measure based on set observational criteria, in this study, undertaken by nurses. In the ward environment, nursing activities are dynamic, there are shift changes and sometimes staff shortages meant casual contract nursing staff were caring for GEM patients. For these reasons, there is the potential for subtle changes and improvements in participants’ behaviour to not be identified in the change of staff over the day/participant’s admission, and thus not captured and accounted for in the data. A further limitation, regarding PAS used in this feasibility trial, was that agitation was scored as a composite measure of all four domains, rather than examining individual PAS domains. Potentially, there could be trends or subtle effects of music on domains that are currently “hidden” within this sole composite PAS. However, as the objective was to assess for feasibility of trial method and processes, there was no impetus to investigate individual domains. Moreover, where PAS was indicated at “0,” it was unclear whether patients were simply not agitated, asleep or sedated secondary to psychotropic medication administration. The PAS nursing notes were relied upon to give investigators this information, but this was not consistently indicated. Additionally, there is the question of whether PAS will be as reliably recorded if a full-scale study were to eventuate, considering that PAS was used as a valid convenience measure of agitation in this pilot for its concurrent use in a separate clinical trial.

Further limitations include the accuracy of other recorded data, including the timing of PRN medication and regular psychotropic medications given in relation to PAS data, which could have affected the agitation scores reflected in the PAS. Moreover, according to the research protocol, those in the intervention arm were able to have music on a PRN basis in addition to the regular 15:00–16:00 period. This was not consistently indicated on the individual patient PAS spreadsheet, meaning an accurate assessment on cumulative music exposure in relation to PAS trends could not be made.

Additionally, the CGI was a potential source of bias due to the difficulty of blinding groups. This may have unconsciously influenced clinicians to score more favourably post-intervention if they identified a patient as having received the music intervention during the trial (92% of intervention group patients vs. 44% of control group patients were rated as “minimally improved” or “much improved”). Additionally, CGI would be a difficult measure to objectively base improvements in clinical condition owing solely to the music intervention because the patient’s condition may have been improving due to appropriate treatment, or the natural course of illness.

### 4.3. Generalisability

Modifications can be made to the study protocol to improve accessibility. One change is broadening inclusion criteria to include all patients with cognitive impairment, as was suggested in interviews. This may assist in recruiting more participants, which may increase the power of the study when assessing intervention efficacy in a larger trial.

Additionally, there are potential environmental modifications that, if made, could optimise the management and capacity of this GEM unit to facilitate the intervention. These include a quieter ward environment (i.e., use of side rooms to minimise background noise and reduce distraction), and recruiting and training additional hospital volunteers, allied health and nursing assistants to facilitate the music intervention with participants to allow nurses to conduct usual duties without feeling impoverished for time.

Furthermore, collating and utilising more data in future studies will provide more variables to use in analysis, which may increase the fit of statistical models used to better explain the data yielded. Specifically, this could mean analysing 1-hourly PAS scores instead of 2-hourly, collecting additional demographic data (i.e., presenting complaint/reason for GEM admission) to increase interpretation of CGI, and documenting psychotropic medications administered or time of sleep in relation to an individual’s PAS score. Practically, timings for medication and sleep may be documented by nursing notes related to PAS timepoints. A further improvement to data analysis would be assessing each PAS domain, rather than grouping it as a composite score of agitation. Analysing each domain may uncover impacts of music on certain manifestations of agitation that were not elucidated in this study, which would be clinically relevant to managing BPSD.

It is also interesting to consider what the results of a future trial may yield. Interventions in RACF have tended to assess a longer duration of music exposure: 30 min once a week for 10 weeks (9), 30 min alternate days for 4 weeks (17) and 30 min of 30 sessions in 16 weeks (4). Conversely, this pilot investigated a relatively short-term intervention (5 days, minimum of 1 h music exposure/day). Hence, there is a question of whether the “dose” of music investigated in this method was sufficient to elicit and observe a quantifiable response.

## 5. Conclusion

This pilot has been a valuable exploration of the feasibility in delivering a music intervention alongside clinical care in a geriatric unit of a tertiary hospital. Staff impressions of feasibility suggest it is possible to deliver a novel music intervention without compromising on clinical care. Additionally, several considerations regarding study modifications for further research have been demonstrated. To undertake such a trial again and reach a higher number of participants is likely to require more resources, including a dedicated study full time equivalent in addition to other recommendations discussed.

Future studies in this field must keep in mind that music is not an antidote to agitation with a consistently observable effect, because it is influenced by changes in a patient’s clinical situation and subjective experience. Furthermore, cognitive decline is a dynamic process that interacts with affect, mood and longitudinal character traits and preferences, thus: “perhaps changes [due to music] cannot be sustained as the dementia becomes more severe. [However] the intervention might still be deemed worthwhile if it improved the person’s quality of life, even temporarily” (10, 32). Such consideration is noteworthy when investigating the effect of subjective music on equally subjective people.

The results of this feasibility trial lean towards more extensive and rigorous investigation of the impact of a PMLI as a non-pharmacological intervention for BPSD, with a focus on efficacy, in a hospital context.

## Data availability statement

The raw data supporting the conclusions of this article will be made available by the authors, without undue reservation.

## Ethics statement

The CALHN Human Research Ethics Committee reviewed and approved this study involving human participants. The participants, or their next of kin, provided written informed consent to participate in this study.

## Author contributions

SL and JM contributed to the conception and design of the study. LC performed the statistical analysis and wrote the statistics section of the manuscript. SL wrote the first total draft of the manuscript. All authors contributed to the article and approved the submitted version.

## Funding

The Hospital Research Foundation Group, South Australia, awarded the research team $10 000 AU for this pilot study.

## Conflict of interest

The authors declare that the research was conducted in the absence of any commercial or financial relationships that could be construed as a potential conflict of interest.

## Publisher’s note

All claims expressed in this article are solely those of the authors and do not necessarily represent those of their affiliated organizations, or those of the publisher, the editors and the reviewers. Any product that may be evaluated in this article, or claim that may be made by its manufacturer, is not guaranteed or endorsed by the publisher.
